# Supplementary figures and images for: Microbial interactions from a new perspective: reinforcement learning reveals new insights into microbiome evolution
Source: Bioinformatics. 2024 Jan 11;40(1):btae003. doi: 10.1093/bioinformatics/btae003 (PMC10799744; doi:10.1093/bioinformatics/btae003)

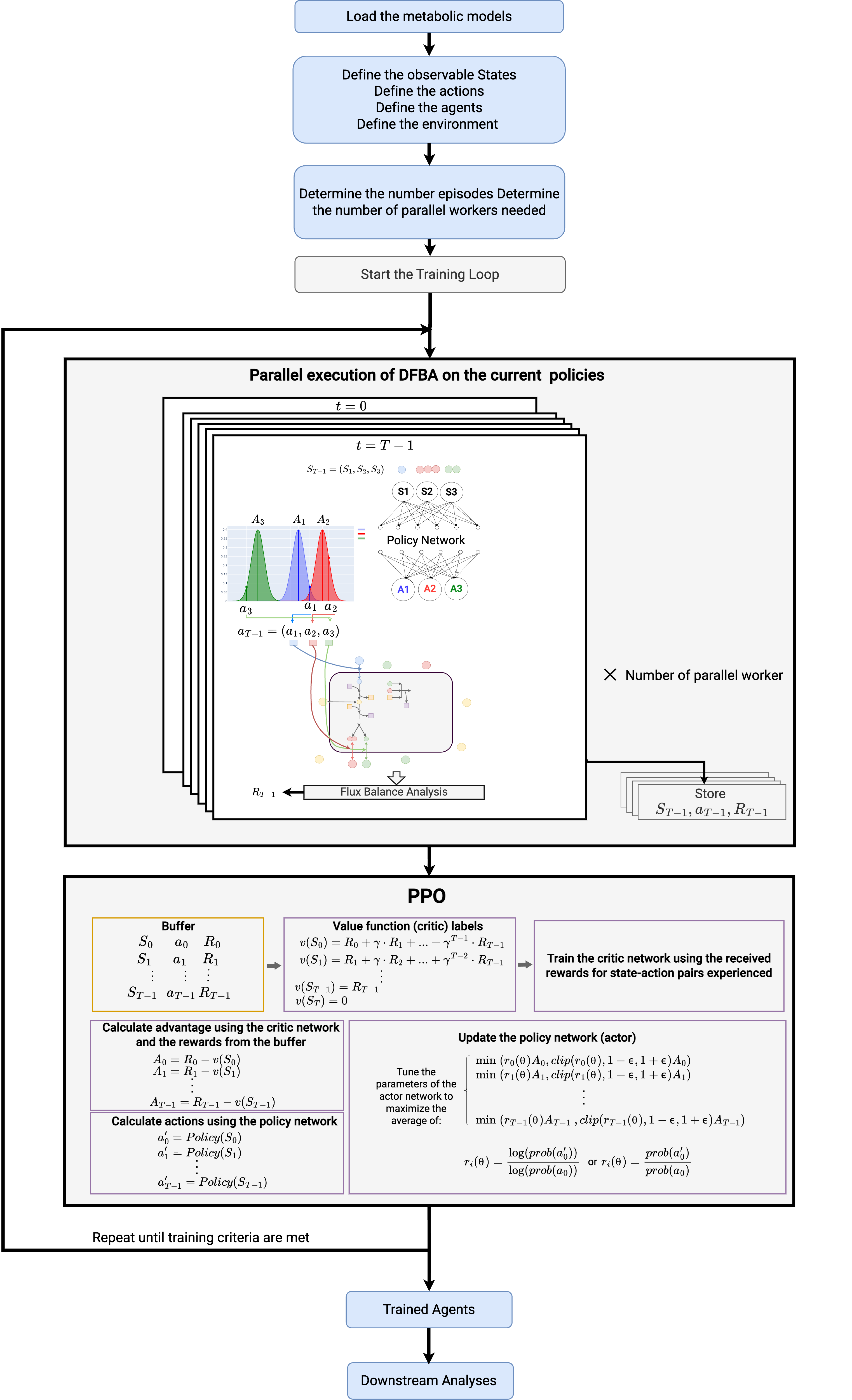

Supplement: btae003_Supplementary_Data [file btae003_supplementary_data.zip › SF1.png]

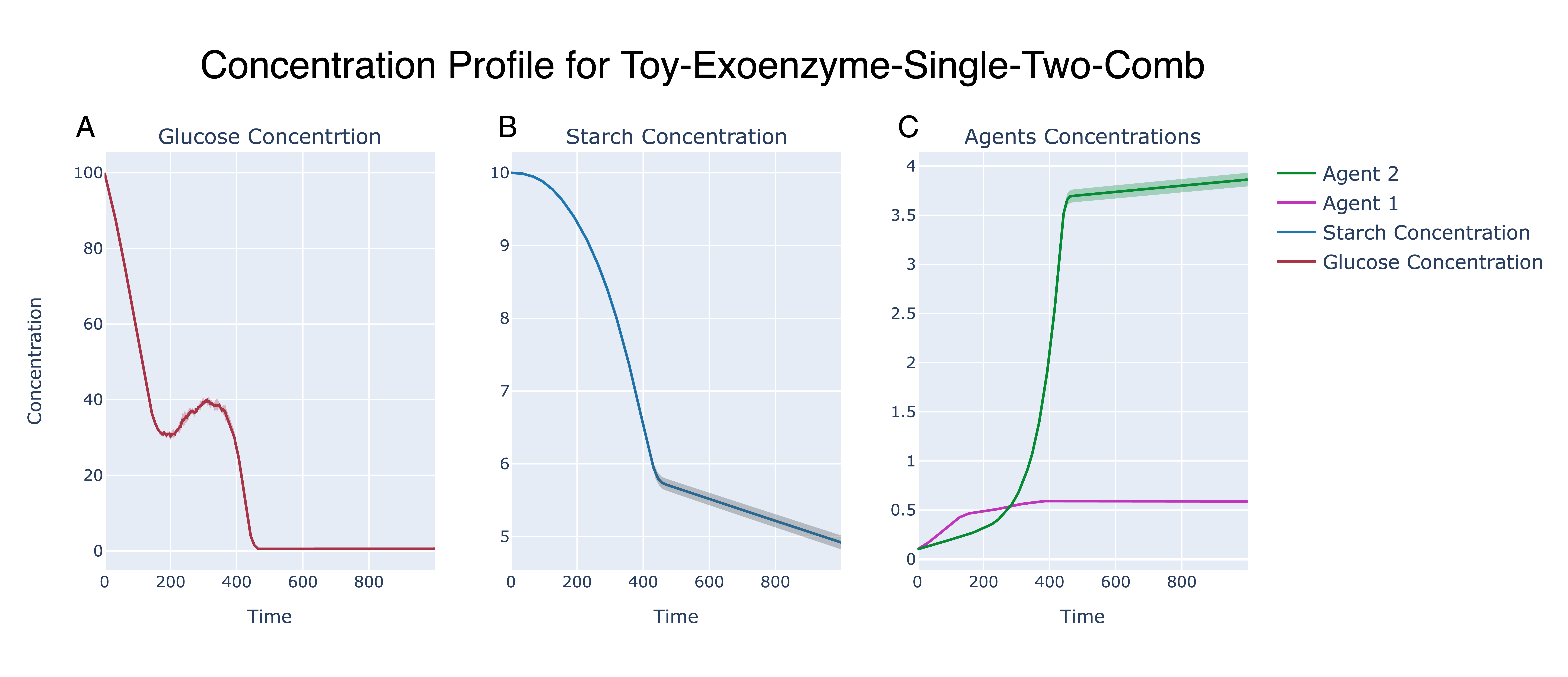

Supplement: btae003_Supplementary_Data [file btae003_supplementary_data.zip › SF2.png]
